# Congenital Central Hypothyroidism due to a Homozygous Mutation in the *TSH*β** Subunit Gene

**DOI:** 10.1155/2011/369871

**Published:** 2011-12-21

**Authors:** Sarah Catharina Grünert, Miriam Schmidts, Joachim Pohlenz, Matthias Volkmar Kopp, Markus Uhl, Karl Otfried Schwab

**Affiliations:** ^1^Center for Pediatrics and Adolescent Medicine, University Hospital Freiburg, 79106 Freiburg, Germany; ^2^Department of Pediatrics, Johannes Gutenberg University Medical School, Mainz, Germany; ^3^Department of Pediatric Pulmonology, Clinic for Pediatric and Adolescent Medicine, University Lübeck, Lübeck, Germany; ^4^Department of Radiology, St. Josefskrankenhaus Heidelberg, Freiburg, Germany

## Abstract

Congenital central hypothyroidism (CCH) is a rare condition occurring in 1 : 20000 to 1 : 50000 newborns. As TSH plasma levels are low, CCH is usually not detected by TSH-based neonatal screening for hypothyroidism, and, as a result, diagnosis is often delayed putting affected children at risk for developmental delay and growth failure. We report on a girl with isolated central hypothyroidism due to a homozygous one-base pair deletion (T313del) in exon 3 of the *TSH*β** subunit gene. The molecular genetic and typical radiologic findings are discussed, and a systematic diagnostic workup for congenital central hypothyroidism is proposed. Physicians need to be aware of this rare condition to avoid diagnostic delay and to install prompt replacement therapy.

## 1. Introduction

Congenital central hypothyroidism (CCH, OMIM#275100) is an uncommon variant of congenital hypothyroidism with an incidence of 20.000 to 1 : 50.000 newborns [1]. CCH may occur in isolation or in combination with panhypopituitarism associated with mutations of genes encoding transcription factors involved in pituitary development [2]. The isolated form of CCH is more difficult to detect clinically because additional clinical signs such as persistent hypoglycaemia in case of panhypopituitarism are lacking. Owing to the efficacy of neonatal screening programs which facilitate early diagnosis and prompt initiation of replacement therapy, the prognosis for children with primary congenital hypothyroidism has improved significantly. Unfortunately, most screening programs are based on the detection of elevated TSH levels only to detect children with primary hypothyroidism [3]. As in CCH TSH levels are low, early diagnosis is usually missed in TSH-based neonatal screening putting affected children at risk for mental retardation and severe growth failure.

## 2. Case Report

A female infant, the first child to nonconsanguineous Caucasian parents, was delivered at 38-week gestation by Caesarean section because of cephalopelvic disproportion. No signs of peripartum infection were evident. Her birth weight, length, and head circumference were 2810 g (10th–25th percentile), 50 cm (25th–50th percentile), and 35 cm (50th–75th percentile), respectively. She was initially tachypnoeic, and mild hypoglycaemia (blood glucose 27 mg/dL) was detected on one occasion on day one. Neonatal sepsis was suspected as C-reactive protein concentrations in blood were elevated, and chest radiograph was suggestive of pneumonia. Intravenous piperacillin and netilmicin were begun. Five days later, blood cultures remained negative, and antibiotic treatment was discontinued. Screening for hypothyroidism was considered to be normal as thyrotropin (TSH) was not elevated (0 *μ*U/mL, normal range 0–15 *μ*U/mL).

**Figure 1:**
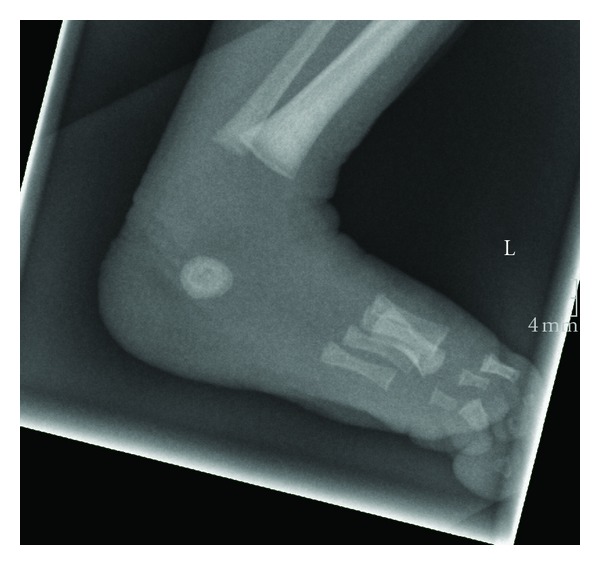
Radiograph of the baby's foot at 20 days of chronological age. Bone age has been classified as 28th–30th gestational week; thus, bone age is significantly retarded (according to the Hernandez/ Erasmie score).

Within the next few days, the patient developed progressive feeding difficulties, muscular hypotonia, and somnolence. Additionally, jaundice was observed (maximum serum bilirubin concentration 15.8 mg/dL). On day 19, thyroid function tests revealed a serum-free thyroxine (fT4) concentration of 0.8 pmol/L (normal range 12–22 pmol/L) while the free L-triiodothyronine (fT3) concentration was 0.4 pmol/L (normal range 3.0–8.4 pmol/L, immunological assay). At the same time, serum TSH was low at 0.05 *μ*U/mL (normal range 0.27–4.2 *μ*U/mL); thus, central hypothyroidism was diagnosed. Her serum thyroglobulin was within the normal range. Panhypopituitarism was excluded by the findings of normal serum levels of ACTH, cortisol, IGF1, IGFBP3, LH, FSH, cortisol excretion in a 24-hour urine, and blood glucose profile. An MRI brain scan was normal. Thyroid ultrasonography revealed a normally located thyroid gland of appropriate size and shape (0.6, normal 0.3–2.3 mL). Isolated central hypothyroidism was confirmed following the detection of elevated prolactin levels (1141 mU/L, normal range 102–496 mU/L). No increase in TSH concentration was noted following stimulation with thyrotropin-releasing hormone (TRH) while prolactin was appropriately stimulated with an increase in concentrations noted from 678 to 954 mU/L. Bone age (Hernandez/Erasmie Score) was significantly delayed by 3 months (Figure 1).

**Figure 2:**
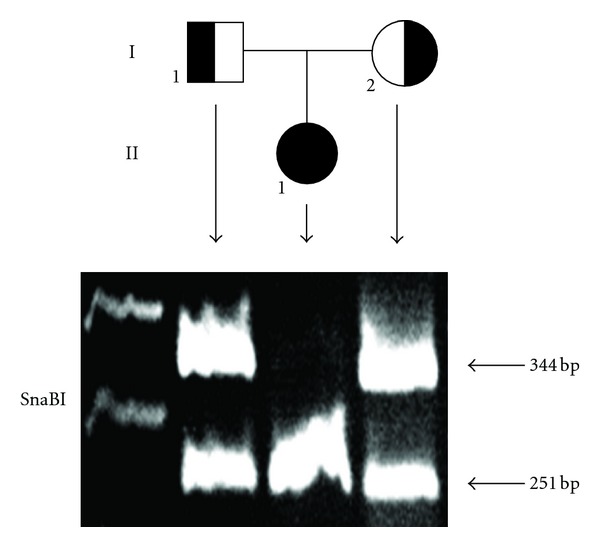
Molecular analysis of genomic DNA identified a homozygous one-base pair deletion (T313del) in exon 3 of the *TSH*β** subunit gene resulting in a frame shift and a premature stop at codon 114. The deletion of nucleotide 313 (T) creates a new restriction site for SnaBI. The allele with the wild-type *TSH*β** fragment remained undigested (344 bp), whereas the mutant allele was cut into two fragments of 251 (as shown in the figure) and 93 bp (not shown). Our patient (II1) is homozygous for this mutation, both parents (I1 and I2) are heterozygous.

Direct sequencing of the entire coding region of the proposita's *TSH*β** gene revealed a homozygous single nucleotide deletion of a T at position 313 in exon 3, resulting in a frame shift mutation with a premature stop at codon 114 (Figure 2). Both clinically unaffected parents were heterozygous for the mutation and had normal thyroid hormone levels.

L-thyroxine replacement therapy was started on day 19 at a starting dose of 50 *μ*g/day. Eleven days later, the dose was reduced to 25 *μ*g/day owing to an elevated fT4 serum concentration. Following the initiation of therapy, both feeding problems and lethargy slowly resolved. At five weeks of age, the patient was well and was subsequently discharged. Since discharge, her three monthly fT3, fT4, and prolactin serum concentrations have been adequately controlled within the upper normal range, while prolactin concentrations initially continued to be elevated. Neurological development was normal at the age of 7 months.

## 3. Discussion

Isolated CCH is a rare variant of congenital hypothyroidism. While only very few cases of a mutation in the *TRH receptor gene* have been published [4], the majority of cases is associated with mutations in the *TSH*β* subunit*[5] (OMIM *188540). TSH is a 28–30 kDa glycoprotein synthesized and secreted by the anterior pituitary gland. Similar to other members of the glycoprotein hormone family, it is a heterotrimeric cystine knot protein consisting of a common *α*-subunit and a specific *β*-subunit. For the *TSH*β** gene, nine different mutations have been identified so far [5, 6], all leading to isolated TSH deficiency. Inheritance is autosomal recessive. The most frequent *TSH*β**-subunit mutation is the 1 bp deletion in codon 105 (c.T313del, p.C105Vfs*) in exon 3 as found in our patient [7]. This mutation leads to a frame shift and a premature stop at codon 114 and predicts the synthesis of a truncated *TSH*β** subunit [7]. As a consequence, this TSH form is usually not detected by routine immunological assays and is biologically inactive at the TSH receptor. A population heterozygote carrier frequency of c.T313del of less than 1 : 170 has been estimated for the German population, and a monophyletic origin is suggested by the results of haplotype analyses in carrier families [8]. While its frequency is too low to warrant population wide neonatal T4 screening, identification and genetic counselling of heterozygous persons in known carrier families are important [5].

Newborn screening has been designed to detect primary hypothyroidism. Since in most screening programs attention is only paid to high TSH levels and fT4 is not measured, patients with CCH are usually missed. Therefore, diagnosis and initiation of thyroid hormone substitution may be delayed in these cases, resulting in developmental delay [5]. Compared to other cases of CCH published so far with diagnosis at the age of several months to years, our patient has been diagnosed early due to distinct clinical signs and postnatal inpatient treatment [5]. Nevertheless, the correct diagnosis has been delayed for a few days due to the attribution of unspecific symptoms such as adynamia, feeding problems, body temperature instability, and prolonged jaundice to neonatal sepsis.

Delayed bone age is a common feature of congenital hypothyroidism and seems to correlate with severity of thyroid dysfunction [9]. Pezzuti et al. reported that in a cohort of 280 Brazilian children with congenital hypothyroidism 32.1% had delayed bone age for their chronological age [10]. In this study, children presenting within the first month of life had significantly more abnormal thyroid function and a higher incidence of retarded bone age in comparison with children older than 30 days (retarded bone age in 41% versus 21%, resp.) [10]. These findings have been confirmed by other studies such as a longitudinal assessment of L-T4 therapy for congenital hypothyroidism in Mexico [11], where patients with delayed bone age at birth had lower T4 and fT4 levels than patients with normal bone age. Delayed bone age at birth reflects prenatal thyroid dysfunction [12] and is usually associated with abnormal neuropsychomotor development within the first year of life, irrespective of other variables related to treatment [9]. Despite significantly delayed bone age at birth, our patient does not show any signs of developmental delay until now. However, psychomotor development will have to be monitored throughout childhood.

**Figure 3:**
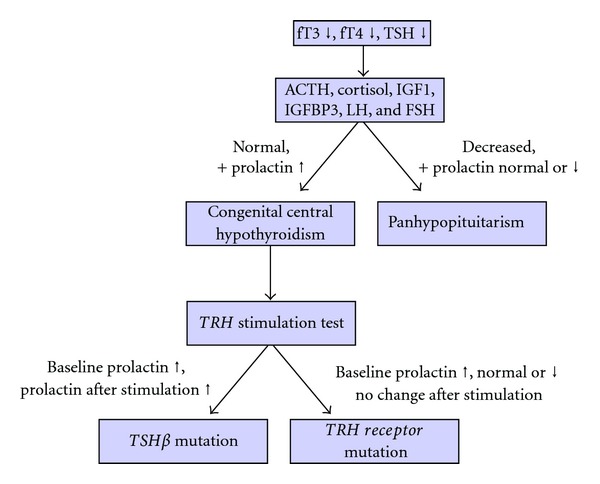
Proposed diagnostic workup in CCH for differentiation between a panhypopituitarism, a *TRH-receptor gene* mutation and a *TSH*β*-gene* mutation. Rare other conditions mimicking CCH, such as hypothyroxinemia due to prematurity, primary hypothyroidism with delayed TSH elevation, and transient CCH must also be ruled out.

To reduce the diagnostic delay in patients with central hypothyroidism, we recommend the following diagnostic workup (Figure 3). As CCH is not detected in TSH-based newborn screening, thyroid hormone levels (fT3, fT4) should be analyzed immediately if hypothyroidism is clinically suspected. Panhypopituitarism can be differentiated from isolated CCH by normal levels of ACTH, cortisol, IGF1/IGFBP3, LH, FSH, and prolactin. For further differential diagnosis, a TRH stimulation test can be helpful. A defective TRH receptor will neither stimulate TSH nor prolactin. However, in case of a *TSH*β** gene mutation, prolactin levels will be normal or high with further increase after TRH stimulation proving normal function of the TRH receptor, but TSH levels remain low. To our knowledge, no genetic TRH deficiency has been reported yet.

## 4. Conclusion

*TSH*β* subunit *gene mutations may affect the structure of TSH resulting in a biologically inactive and immunologically nonreactive protein. Affected infants may be missed in TSH-based newborn screening programs unless attention is paid to abnormally low TSH levels. Physicians need to be aware of this rare condition to avoid diagnostic delay and to install prompt replacement therapy.
